# Differential memory persistence of odor mixture and components in newborn rabbits: competition between the whole and its parts

**DOI:** 10.3389/fnbeh.2014.00211

**Published:** 2014-06-16

**Authors:** Gérard Coureaud, Thierry Thomas-Danguin, Frédérique Datiche, Donald A. Wilson, Guillaume Ferreira

**Affiliations:** ^1^Centre des Sciences du Goût et de l’Alimentation (CSGA), UMR 6265 CNRS, UMR 1324 INRA, Université de BourgogneDijon, France; ^2^Department of Child and Adolescent Psychiatry, New York University Langone School of MedicineNew York, NY, USA; ^3^Nutrition and Integrative Neurobiology Group, INRA UMR 1286Bordeaux, France; ^4^Université de BordeauxBordeaux, France

**Keywords:** *Oryctolagus cuniculus*, newborn, odor mixture, configural perception, stimulus representation, retention, memory persistence

## Abstract

Interacting with the mother during the daily nursing, newborn rabbits experience her body odor cues. In particular, the mammary pheromone (MP) contained in rabbit milk triggers the typical behavior which helps to localize and seize the nipples. It also promotes the very rapid appetitive learning of simple or complex stimuli (odorants or mixtures) through associative conditioning. We previously showed that 24 h after MP-induced conditioning to odorants A (ethyl isobutyrate) or B (ethyl maltol), newborn rabbits perceive the AB mixture in a weak configural way, i.e., they perceive the odor of the AB configuration in addition to the odors of the elements. Moreover, after conditioning to the mixture, elimination of the memories of A and B does not affect the memory of AB, suggesting independent elemental and configural memories of the mixture. Here, we evaluated whether configural memory persistence differs from elemental one. First, whereas 1 or 3-day-old pups conditioned to A or B maintained their responsiveness to the conditioned odorant for 4 days, those conditioned to AB did not respond to the mixture after the same retention period. Second, the pups conditioned to AB still responded to A and B 4 days after conditioning, which indicates stronger retention of the elements than of the configuration when all information are learned together. Third, we determined whether the memory of the elements competes with the memory of the configuration: after conditioning to AB, when the memories of A and B were erased using pharmacological treatment, the memory of the mixture was extended to day 5. Thus, newborn rabbits have access to both elemental and configural information in certain odor mixtures, and competition between these distinct representations of the mixture influences the persistence of their memories. Such effects certainly occur in the natural context of mother-pup interactions and may contribute to early acquisition of knowledge about the surroundings.

## Introduction

In some cases, mixtures of volatile molecules are perceived as a collection of independent, identifiable elements; the perception is then elemental (e.g., Laing and Francis, 1989; Laska and Hudson, 1993; Linster and Cleland, 2004). However, some mixtures induce a configural processing. Then, the mixture gives rise to either a unique and novel odor quality, different from the odor qualities of the elements (robust configural perception; e.g., Smith, 1996; Jinks and Laing, 1999; Kay et al., 2005), or to a novel quality perceived in addition to the qualities of the odorants (weak configural perception; Rescorla, 1973; Kay et al., 2005). Configural odor processing has been described with different mixtures and different approaches in a variety of species from invertebrates to vertebrates, including humans (e.g., Derby et al., 1996; Linster and Smith, 1999; Valentincic et al., 2000; Wise and Cain, 2000; Deisig et al., 2001; Wiltrout et al., 2003; Mandairon et al., 2006; Riffell et al., 2009; Gottfried, 2010; Wilson and Sullivan, 2011). For instance, data in human adults revealed that a mixture of two odorants (AB), one smelling like strawberry (A: ethyl isobutyrate) and the other like caramel (B: ethyl maltol), generates the configural perception of a pineapple odor at a specific ratio of A/B (30/70 v/v; Le Berre et al., 2008, 2010; Barkat et al., 2012). Interestingly, recent results in a newborn mammal, the newborn rabbit, showed similar configural processing abilities with the same AB mixture, at the same ratio. Indeed, after single appetitive conditioning to odorant A or to odorant B by pairing with the mammary pheromone (MP) (naturally contained in rabbit milk and experimentally used as unconditioned stimulus in associative conditioning procedure; Coureaud et al., 2006, 2010), rabbit pups respond to the conditioned element and respectively to the AC or BC mixtures (C: guaïacol). However, they do not respond to the AB mixture. These results suggest that they perceive AB differently from the odors of A and of B, while they perceive AC or BC as the sum of their component odors (Coureaud et al., 2008, 2009a, 2011a). Furthermore, after single conditioning to the AB mixture, rabbit pups respond to the components A and B in addition to AB, indicating that the AB mixture is perceived in a weak configural way (Coureaud et al., 2008). Very recently, combining behavioral approaches with pharmacological tools, we provided another confirmation of this neonatal weak configural perception of AB and demonstrated that rabbit pups memorize the AB configuration independently (at least in part) from the representations of each element. After conditioning to AB, amnesia of A and B did not propagate to AB: pups that did not respond to either A or B still responded to AB (Coureaud et al., in press). Thus, after AB conditioning, distinct elemental and configural memories of the mixture are created.

In the previous studies in newborn rabbits, memories of the elements A and B or of the AB configuration were evaluated 24 or 48 h after conditioning, which are the optimal delays for responsiveness to a stimulus conditioned by single pairing with the MP (Coureaud et al., 2006). However, to date, whether memory persistence of the configural odor of the AB mixture differs from that of the odorants A and B (also detected in the mixture) had never been evaluated; here, we hypothesized that they could be distinguished. This issue constituted the first and main goal of our study. Besides understanding early memory and perception in the rabbit, this evaluation could more broadly help provide additional and original information on the general topic of odor object perception in mammals. Indeed, as said above, a perceptual match has been evidenced for the AB mixture between rabbit pups and human adults, which suggests a relative conservation in the processing of certain odor mixtures across species.

Our second goal aimed to assess whether memory retention of the configural AB odor and of the elemental odors of A and B depends on the age of the pups at conditioning. Indeed, it is known in newborn mammals (especially in altricial mammals, which develop very rapidly) that the meaning acquired by a conditioned odorant can change from one day to another (e.g., Barr et al., 2009; Sullivan and Holman, 2010). In previous experiments, conditioning was mainly performed in 2-day-old rabbit neonates. In the present study, conditioning was therefore conducted either 1 or 3 days after birth to determine the influence of development on the perception and memory of a complex odor stimulus.

These two issues were evaluated by taking advantage of the configural nature of the previously described AB mixture. To date, this is the only mixture which has been extensively characterized regarding its perceptual properties (weak configural perception) in the rabbit both in terms of behavior (Coureaud et al., 2008, 2009a, 2011a; Sinding et al., 2011), memory (Coureaud et al., in press) and brain processing (Schneider et al., in preparation). To that goal, we conditioned either 1- or 3-day-old pups to simple odorants A or B, or to their binary mixture, and assessed the retention of the conditioned stimuli from 24 to 96 h after conditioning using independent groups for each stimulus and each retention interval (Experiment 1). Pups conditioned to the AB mixture were also tested for their responsiveness to A and to B with the aim to evaluate, by means of a within-subject analysis, whether responsiveness to AB differed over time from that of the single odorants (Experiment 2). Finally, as differences appeared in the responsiveness to the mixture and to its odorants in the first two experiments, we determined whether competition occurred between the memory of AB and the respective memories of A and B created during conditioning to the whole mixture (Experiment 3). Indeed, interference and competition between different associative memories have been classically reported (e.g., Eisenberg et al., 2003; Suzuki et al., 2004; Bradfield and Balleine, 2013) and might contribute to the present results. Therefore, we either prevented the formation of the configural memory by successive conditioning to the single odorants only, or erased the elemental memory after initial conditioning to the AB mixture followed by separate reactivations of odorants A and B and amnesia-inducing anisomycin (AN) injection (Coureaud et al., 2009b, 2011b, 2013). We then evaluated whether these two procedures influenced the responsiveness to the AB mixture after a long retention interval.

## Materials and methods

### Animals and housing conditions

Males and females New-Zealand rabbits *Oryctolagus cuniculus* (Charles River strain, L’Arbresle, France) from the Centre de Zootechnie of the University of Burgundy (Dijon) were kept in individual cages. A nest box (0.39 × 0.25 × 0.32 m) was added on the outside of the pregnant females’ cages 2 days before delivery (day of delivery was day 0; d0). To equalize pups’ nursing experience, all females had access to their nest between 11:30–11:45 a.m. This procedure allowed females to follow the brief (3–4 min) daily nursing of the species (Zarrow et al., 1965). Animals were kept under a constant 12:12 light:dark cycle (light on at 7:00 a.m.) with ambient air temperature maintained at 21–22°C. Water and pelleted food (Lapin Elevage 110, Safe, France) were provided *ad libitum*. In the study, 524 newborns (from 106 litters) were used.

The study was carried out under the local, institutional and national rules (French Ministries of Agriculture, and of Research and Technology) regarding the care and experimental use of the animals. All experiments were conducted in accordance with ethical rules enforced by French law, and were approved by the Ethical Committee for Animal Experimentation (no. 2406).

### Odorants

The stimuli consisted of 2-methylbut-2-enal (the Mammary Pheromone, MP, CAS# 497-03-0), ethyl isobutyrate (odorant A, CAS# 97-62-1), ethyl maltol (odorant B, CAS# 4940-11-8) for pure components, and of the AB mixture. This mixture included 0.3 × 10^−5^ and 0.7 × 10^−5^ g/ml of components A/B; the 30/70 v/v ratio elicits the configural perception of a pineapple odor in human adults due to blending properties (Le Berre et al., 2008, 2010; Barkat et al., 2012), and weak configural perception in newborn rabbits (Coureaud et al., 2008, 2009a, 2011a; Sinding et al., 2011).

The MP allowed inducing the learning by the pups of odorant A, odorant B, or the AB mixture through associative conditioning (see below). It was used at 10^−5^ g/ml, a concentration known to be highly efficient to promote conditioning (Coureaud et al., 2006). Thus, the A-MP and B-MP blends were prepared at a final concentration of 10^−5^ g/ml of each constituent. The AB-MP blend included 1 × 10^−5^ g/ml of MP and 0.3 and 0.7 × 10^−5^ g/ml of odorants A and B.

Single odorants A and B were also used in the reactivation procedure (Experiment 3b) at a concentration of 10^−5^ g/ml.

In all experiments, we deliberately kept constant the overall concentration of the different stimuli (single odorants or AB mixture and blends; 10^−5^ g/ml) and maintained this constancy between conditioning and testing to avoid direct influence of the concentration on our results and to focus on the influence of complexity (single odorant vs. mixture).

All the odorants were purchased from Sigma-Aldrich (Saint-Quentin Fallavier, France) and all the final solutions were prepared in a solvent composed of 0.1% of ethanol (anhydrous, Carlo Erba, Val de Reuil, France) and 99.9% of MilliQ water (Millipore, Molsheim, France).

### Odor conditioning, reactivation and pharmacological treatment

Conditioning sessions were run on day 1 or 3 after birth in an experimental room close to the breeding room. The pups from an individual litter were transferred by groups of 5 (usual case) or 4 (Experiment 3b) into a box maintained at room temperature. The MP-induced conditioning was run following a procedure previously described (e.g., Coureaud et al., 2006, 2008, 2009a,b, 2013; Sinding et al., 2011; Charra et al., 2013). This procedure offered the advantage of being extremely rapid (single trial) and appetitive (thus avoiding the possible modulation of responsiveness due to the negative emotional state that may occurred after aversive conditioning).

In most cases, just before the conditioning session, 4 ml of the A-MP (Experiment 1), B-MP (Experiment 1) or AB-MP (Experiments 1, 2 and 3b) blends were pipetted on a pad (19 × 14 cm, 100% cotton) then held 2 cm above the pups for 5 min. The conditioning occurred 1 h before the daily nursing (10:30 a.m.) to equalize the pups’ motivational state and limit the impact of satiation on behavioral responses (Montigny et al., 2006). Two minutes after the end of the conditioning, the pups were individually marked with ink and returned to their nest. The box containing the pups was rinsed with alcohol and with distilled water after each conditioning session.

In one group (Experiment 3a), the procedure was the same except that pups were conditioned successively to odorants A and B: they were exposed to the A-MP blend for 2.5 min (for the half of the pups, randomly chosen, and B-MP for the other half), then transferred to a second box in which they remained non stimulated for 1 min before being exposed to the B-MP (or A-MP) blend for 2.5 min.

Finally, in two other groups (Experiment 3b), 24 h after the conditioning to the AB mixture, the memory of pups was reactivated by exposure to odorant A then odorant B in half of the pups (or conversely in the other half) following the same procedure than above (2.5 min per odorant, delay inter-stimulation: 1 min). Immediately after reactivation, anisomycin (AN; Sigma-Aldrich) was injected to the half of the pups (42 mg/kg, i.p.) after dilution in 0.9% NaCl solution and adjustment to pH 7.2 with 1N HCl. The AN was used after reactivation performed at 24 h as we have previously demonstrated the effectiveness of this procedure in erasing memory of odor element(s) in newborn rabbits (Coureaud et al., 2009b, 2011b, 2013, in press). Control for the effect of AN injection was carried out with the other half of animals, which received saline 0.9%. As in other studies with newborn or adult mammals (e.g., Davis and Squire, 1984; Gruest et al., 2004; Desgranges et al., 2008) we considered that AN in newborn rabbits may induce a real amnesia and not a perturbation in responsiveness due to an aversive effect. Pups were returned to the nest just after AN or saline injection.

### Behavioral assay

The behavioral assay occurred 24, 48, 72 or 96 h after the conditioning, i.e., on days 2, 3, 4 or 5 when the conditioning occurred on day 1, and on days 4, 5, 6 or 7 when it happened on day 3. The assay was run in the experimental room previously used for conditioning and reactivation, and happened also 1 h before the daily nursing to limit the impact of satiation on motivation and behavioral responsiveness (Montigny et al., 2006). It consisted of an oral activation test during which a pup was immobilized in one gloved hand of the experimenter, its head being left free. The odor stimulus was presented for 10 s with a glass rod 0.5 cm in front of the nares (e.g., Coureaud et al., 2006, 2008, 2011a, 2013). A test was positive when the stimulus elicited head-searching movements (vigorous, low amplitude horizontal and vertical scanning movements displayed after stretching towards the rod) usually followed by grasping movements (labial seizing of the rod extremity). Non-responding pups displayed no response except sniffing. Pups were tested in groups of 4 or 5 (same groups than during the conditioning), and only once, on a given day (i.e., different groups were tested on different days).

In Experiment 1, the pups were tested for their responsiveness to one stimulus only, except those which were conditioned to the AB mixture. The latter were indeed tested to AB but also (these results are the results of Experiment 2) to odorants A and B. In Experiment 3, the pups were tested to A, B and AB. Successive testing involved the presentation of a first stimulus to a pup, then a second stimulus to another pup, and so on with an inter-trial interval of 60 s. The order of stimuli presentation was systematically counterbalanced from one to another pup. If a pup responded to a stimulus, its nose was softly dried before the next stimulation. The pups were immediately reintroduced in their nest after testing.

### Statistics

Each group was composed of 18–20 pups except the groups of Experiment 3b (with saline and AN injection; *n* = 11–12 pups). The proportions of pups responding during the behavioral assay were compared using either the χ^2^-test of Pearson (with Yates correction when necessary) when the groups were independent (i.e., distinct groups tested for their response to a same stimulus on different days, or to different stimuli on a given day), or the Cochran’s *Q*-test when the groups were dependent (i.e., pups from a same group tested for their response to the three stimuli). When the Cochran’s *Q*-test was significant, proportions of responding pups were compared 2 × 2 by the χ^2^-test of McNemar. Degrees of freedom are indicated when >1. Data were considered as significant when the two-tailed test ended with *p* < 0.05.

## Results

### Experiment 1. Retention of A, B and AB after their respective learning on day 1 or day 3

To compare the retention of the odors of odorant A, odorant B and of the AB configuration, four groups of pups were conditioned to A on day 1, four other groups to B and four other groups to AB (*n* = 20 pups/group, one pup died in one of the groups; all groups were independent). Each group was tested for its behavioral responsiveness to the conditioned stimulus at one single time point only: 24, 48, 72 or 96 h after the conditioning. The same experiment was conducted with 16 other independent groups of pups conditioned on day 3 instead of day 1 (*n* = 20/group, one pup died in two groups and two pups in another group).

**Figure 1:**
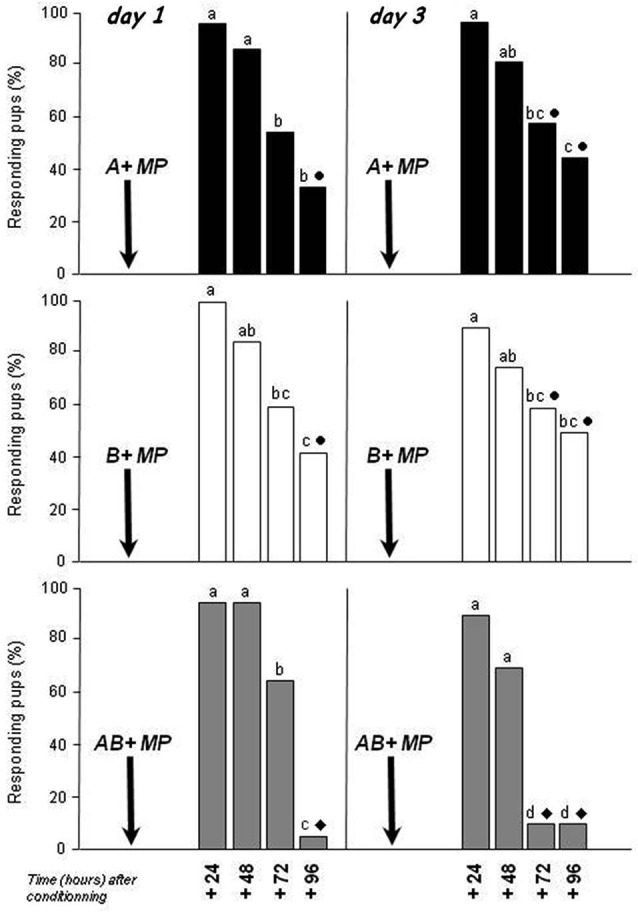
**Proportions of rabbit pups responding in an oral activation test to odorant A (ethyl isobutyrate; black bars), odorant B (ethyl maltol; white bars) or the AB mixture (gray bars) 24, 48, 72 and 96 h after conditioning to one of the stimuli**. Conditioning occurred on day 1 or day 3 by pairing with the mammary pheromone (MP). Independent groups of pups (*n* = 18 to 20/group) were tested on each day. Distinct letters indicate significant differences in responsiveness over time between pups conditioned to the same stimulus, and distinct symbols indicate differences on a given period between pups conditioned to different stimuli.

After conditioning on day 1 (Figure 1, left column), the responsiveness of pups to the conditioned stimulus decreased over time whatever the nature of the stimulus (i.e., in pups conditioned to A, or B or AB; χ^2^ > 19.37, *df* = 3, *p* < 0.001 in the three situations). In pups conditioned to A, the responsiveness to the odorant was maximal and similar 24 and 48 h after the conditioning (>85%; χ^2^ < 1, *p* > 0.05), lower at 72 h (55%; χ^2^ > 4.3, *p* < 0.05 compared to 24 and 48 h) and 96 h (35%; χ^2^ > 8.4, *p* < 0.01 compared to 24 and 48 h), and not different between 72 and 96 h (χ^2^ = 1.6, *p* > 0.05). Regarding the pups conditioned to B, the pattern of responsiveness was nearly the same. The responsiveness was maximal and equivalent at 24 and 48 h (>85%; χ^2^ = 1.4, *p* > 0.05), lower at 72 h (60%; comparison with 24 h: χ^2^ = 7.6, *p* < 0.01, with 48 h: χ^2^ = 3.1, *p* = 0.07) and 96 h (42.1%; comparison with 24 and 48 h: χ^2^ > 6.03, *p* < 0.01), and similar between 72 and 96 h (χ^2^ = 1.2, *p* > 0.05). After conditioning to the AB mixture, the pups highly and similarly responded to AB 24 and 48 h later (95%) and less at 72 h (65%; comparison with 24 and 48 h: χ^2^ = 3.9, *p* < 0.05). At 96 h, only 5% of the pups responded, a level which was lower compared to 24, 48 and 72 h (χ^2^ > 18.6, *p* < 0.001). Interestingly, whereas the responsiveness of the three categories of pups (conditioned to A, B and AB) did not differ at 24, 48 and 72 h (between categories comparisons: χ^2^ < 1.29, *df* = 2, *p* > 0.05), at 96 h the pups conditioned to AB responded less to this stimulus than the pups conditioned to A or B (χ^2^ = 7.74, *df* = 2, *p* < 0.05; comparisons AB vs. A or B: χ^2^ > 3.91, *p* < 0.05; comparison A vs. B: χ^2^ < 0.5, *p* > 0.05).

After conditioning on day 3 (Figure 1, right column), the retention of the acquired stimulus changed over time independently of its nature (χ^2^ > 8.28, *df* = 3, *p* < 0.05 in the three situations). In pups conditioned to A or B, the responsiveness was maximal and close at 24 and 48 h (>75%; χ^2^ < 0.9, *p* > 0.05 for A or B comparisons), lower at 72 h (around 60%) compared to 24 h (χ^2^ > 4.8, *p* < 0.05 for A or B), and lower at 96 h compared to 24 and 48 h for A (45%; χ^2^ > 5.2, *p* < 0.05) or compared to 24 h for B (50%; χ^2^ = 7.4, *p* < 0.001). The responsiveness was similar between 96 and 72 h both in pups conditioned to A and in pups conditioned to B (χ^2^ < 0.6, *p* > 0.05). In pups conditioned to AB, the responsiveness to AB was strong and equivalent at 24 and 48 h (>70%; χ^2^ = 1.4, *p* < 0.05), but it became extremely weak as soon as 72 h (<10.5%; comparisons between 72 or 96 vs. 24 or 48 h: χ^2^ > 11.8, *p* < 0.001). While the responsiveness to the conditioned stimulus was equivalent between pups conditioned to A, B or AB at 24 and 48 h (χ^2^ < 0.5, *df* = 2, *p* > 0.05), it was lower in pups conditioned to AB than to A or to B at 72 and 96 h (χ^2^ > 8.91, *df* = 2, *p* > 0.05; A or B vs. AB: χ^2^ > 4.88, *p* < 0.05; A vs. B: χ^2^ < 0.5, *p* > 0.05).

Thus, whatever the information that was learned (A, B, or AB) on either day 1 or 3, its retention by the pups decreased over the 4 post-conditioning days. However, the retention of the AB mixture or of its odorants was not the same: all the pups stopped responding to the mixture 72 and/or 96 h after conditioning while a significant proportion of them (>35%) were still responding to the odorant they have previously learned. Besides the time of memory testing, the animal’s age at conditioning also influenced the retention of AB memory: pups conditioned to AB at day 3 stopped responding earlier than those conditioned at day 1 (72 h vs. 96 h). This effect of age was not observed for A and B memories when comparing conditioning at day 1 and 3.

### Experiment 2. Retention of A, B and AB after learning of AB on day 1 or 3

Results of Experiment 1 showed clear differences in the retention of the odors of A, B and AB when each odorant or the mixture were learned separately by different pups, suggesting that the memory retention of the binary mixture is weaker compared to the retention of its components. Here, we assessed whether similar results could be obtained at the individual level (within-subject design), namely after conditioning to AB (during which acquisition of the A and B elements and of the AB configuration happens; Coureaud et al., 2008, 2009a, 2011a; Sinding et al., 2011) and during successive testing to the three stimuli. To that goal, the pups conditioned to the AB mixture on day 1 or 3 in Experiment 1 (4 independent groups/day) were tested for their response to A and to B, in addition to AB, at one single time point only, i.e., either at 24, 48, 72 or 96 h. According to the results of Experiment 1, it was expected that the retention of the configuration would be shorter than the retention of each element that compose the mixture.

**Figure 2:**
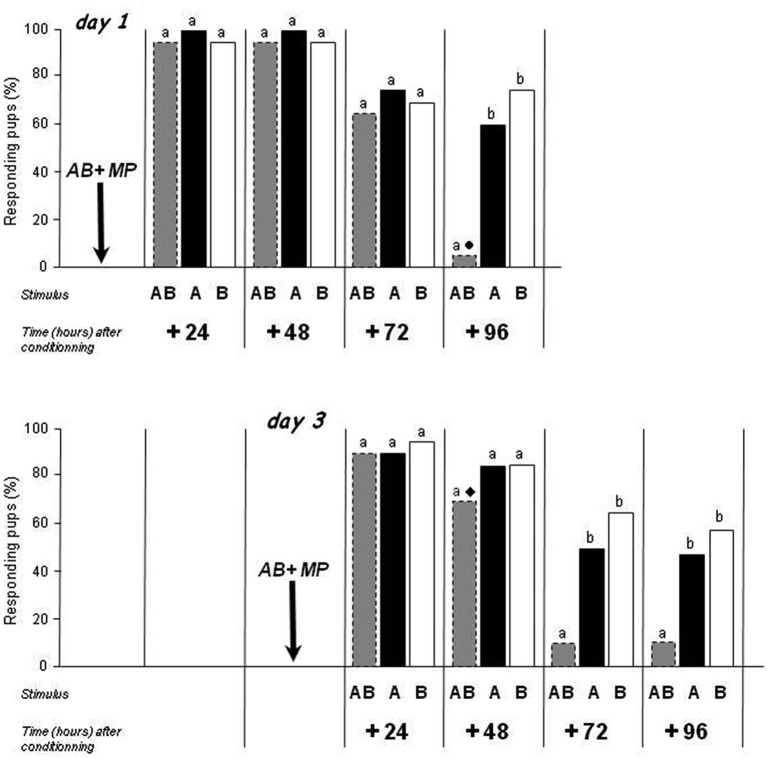
**Proportions of rabbit pups responding in an oral activation test to odorant A (ethyl isobutyrate), odorant B (ethyl maltol) and the AB mixture 24, 48, 72 and 96 h after conditioning to that mixture**. Conditioning occurred on day 1 or 3 by pairing with the MP. Independent groups of pups (*n* = 19 or 20/group) were tested on each day. Distinct letters indicate significant differences in responsiveness of the same pups to the three stimuli presented at a given time, and distinct symbols indicate differences in responsiveness to a same stimulus of different pups conditioned on days 1 or 3 and tested during the same postnatal day (dotted bars correspond to results already presented in Figure 1).

After conditioning to AB on day 1 (Figure 2, upper part), the pups strongly and similarly responded to AB, A and B at 24 and 48 h (>95%; *Q* < 1.1, *df* = 2, *p* > 0.05). At 72 h, the responsiveness decreased for AB (Exp. 1) but also for A and for B (responsiveness to A or B at 72 vs. 24 or 48 h: χ^2^ > 3.9, *p* < 0.05); it remained however similar between AB, A and B (65–75%; *Q* = 1.2, *df* = 2, *p* > 0.05). At 96 h, the responsiveness to A and to B was still around 60 and 75% (thus lower than at 24 and 48 h for A: χ^2^ = 7.6, *p* < 0.01, equivalent for B: χ^2^ = 3.1, *p* = 0.07, and similar for A and for B compared to 72 h: χ^2^ < 1.02, *p* > 0.05). Strikingly, in the same animals, while responsiveness to A and B was relatively maintained, the responsiveness to AB was dramatically reduced (5%; *Q* = 23.3, *df* = 2, *p* < 0.001; AB vs. A or B: McNemar χ^2^ > 9.09, *p* < 0.01; A vs. B: McNemar χ^2^ = 1.3, *p* > 0.05).

Similar results were observed after conditioning on day 3 (Figure 2, lower part), though with a slightly different time course. After conditioning to AB on day 3, the responsiveness of pups was high and similar to AB, A and B 24 h later (>90%; *Q* = 3, *df* = 2, *p* > 0.05) and 48 h later (70–85%; *Q* = 3, *df* = 2, *p* > 0.05). At 72 and 96 h, the responsiveness to A and B remained around 50 and 60% (lower than at 24 h, χ^2^ > 5.6, *p* < 0.01, but not different than at 48 h, χ^2^ < 2.1, *p* > 0.05), but the responsiveness to AB was weaker (<10.5%; for the two periods: *Q* > 14.88, *df* = 2, *p* < 0.001; AB vs. A or B: McNemar χ^2^ > 5.14, *p* < 0.05; A vs. B: McNemar χ^2^ < 1.3, *p* > 0.05).

Thus, in line with the data from Experiment 1 obtained with independent groups of pups, the responsiveness of newborn rabbits to the mixture and to its components decreased over time after conditioning to the mixture and individual testing to the three A, B and AB stimuli: it disappeared earlier for the mixture than for the odorants, and earlier after conditioning on day 3 than on 1.

Moreover, on postnatal day 5, the proportion of pups still responding to AB was higher in pups conditioned on day 3 than in pups conditioned on day 1 (70 vs. 5%; χ^2^ = 15.3, *p* < 0.001) while the responsiveness to A or to B was similar (60–85%; χ^2^ < 0.5, *p* > 0.05). This suggests that the difference observed for AB was the consequence of memory processes and not of pure developmental effects.

### Experiment 3. Competition between the memories of the elements and of the configuration

According to the results of Experiments 1 and 2, newborn rabbits have a distinct memory and retention of the AB mixture and of its components. After acquisition of the AB mixture, this difference in terms of retention could be due to a competition between the memory of each odorant and the memory of the AB configuration (since the pups perceived both the odorants and the configuration in the weak configurally processed AB mixture). To evaluate this hypothesis, two procedures were followed.

**Figure 3:**
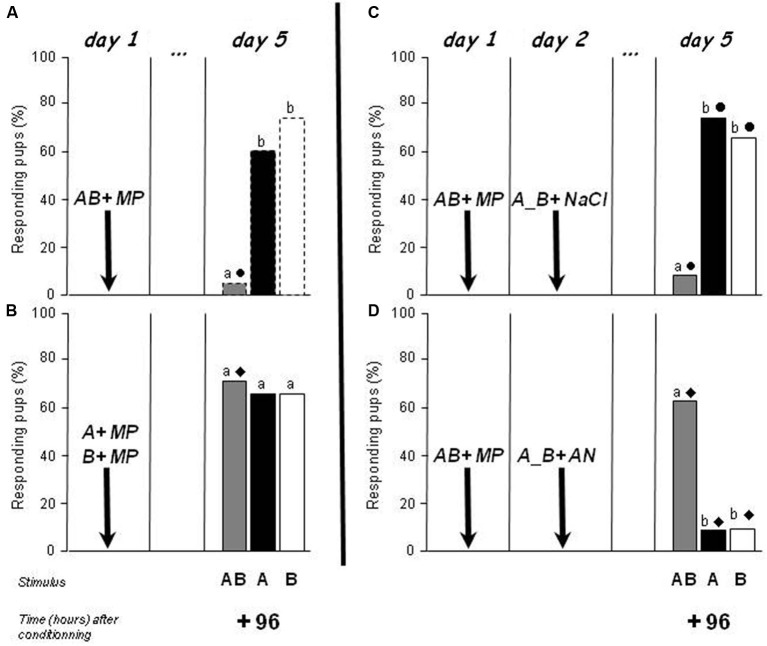
**Proportions of rabbit pups responding in an oral activation test to odorant A (ethyl isobutyrate), odorant B (ethyl maltol) and the AB mixture 96 h after conditioning to AB on day 1 (Figures 3A,C,D, *n* = 20, 12 and 11 pups, respectively), or after successive conditioning to A then to B on day 1 (Figure 3B, *n* = 18 pups)**. Conditioning was performed by pairing with the MP. In Figures 3C,D, the pups were separately exposed to odorants A and B the day after the conditioning and immediately injected either with saline (NaCl) or anisomycin (AN). Distinct letters indicate significant differences in responsiveness to the three stimuli in the same pups, and distinct symbols indicate differences in responsiveness to the same stimulus in pups differently conditioned (**3A** vs. **3B**) or differently treated the day after conditioning (**3C** vs. **3D**) (dotted bars correspond to results already presented in Figure 2).

The first procedure (Experiment 3a) attempted to prevent the creation of a memory for the configuration. In a previous paper (Coureaud et al., 2008), we showed that after successive conditioning to odorant A then odorant B on day 1, the pups responded 24 h later to the AB mixture (they did not display such a response after conditioning to a single odorant). Here, this paradigm was repeated but with a test of responsiveness 96 h later. This group (*n* = 20 pups, two of them died, results concerned 18 pups) was compared to the group conditioned to the whole mixture on day 1 and tested on day 5 (+96 h) in Experiment 2 (i.e., a group that learned the AB configuration). Whereas the conditioning to the AB mixture was followed by an absence of responsiveness to AB but not to A and B at 96 h (Figure 3A, same results as in Figure 2), the successive learning of A and B induced a high and similar level of responsiveness to both the mixture and the individual odorants (66.6–72.2%; *Q* = 1, *df* = 2, *p* > 0.05) (Figure 3B). The responsiveness to AB was therefore higher in the situation of successive learning of the elements than after learning of the mixture (72.2 vs. 5%; χ^2^ = 15.6, *p* < 0.001). The responsiveness to the odorants was similar in the two experimental conditions (60–75%; χ^2^ < 0.5, *p* > 0.05). Therefore, an absence of perception of the AB configuration during conditioning, due to separate and successive conditioning to odorants A and B, was followed by the maintenance of responsiveness to the AB mixture 4 days later.

The second procedure (Experiment 3b) attempted to promote the creation of memories to both the configuration and the elements after AB conditioning, and to make the pups rapidly amnesic of the individual odorants’ odor. We hypothesized that in this situation, the responsiveness to AB could be maintained 4 days after the conditioning because of the absence of competition between the configural and elemental memories. Thus, two independent groups of newborns (*n* = 12/group, 4 pups/litter, 2 pups/litter/group) were conditioned on day 1 to the AB mixture, and reactivated the day after (day 2) by separated exposure to each individual odorant (A then B for half of the pups in each group, B then A for the other half). Immediately after memory reactivation, the first group was injected with saline, while the other group received an AN injection inducing amnesia. The pups were all tested for their responses to AB, A and B on day 5, 96 h after the conditioning (72 h after the reactivation). Blocking memory for the individual odorants induced major differences in mixture memory between the groups. The pattern of response in the saline-treated group (Figure 3C) was the same as in pups from previous experiments which were not manipulated on day 2 (Figure 3A): they did not respond to AB but still displayed a high and similar level of responsiveness to A and to B (8.3 vs. 75.0 vs. 66.6%, respectively; *Q* = 12.6, *df* = 2, *p* < 0.01; AB vs. A or B: McNemar χ^2^ > 5.1, *p* < 0.05; A vs. B: McNemar χ^2^ < 0.5, *p* > 0.05). In contrast, newborns treated with AN (1 pup died, *n* = 11 for this group in the results) showed a completely reversed pattern of responsiveness to the mixture compared to the elements (*Q* = 10.3, *df* = 2, *p* < 0.01) (Figure 3D): they responded strongly to the mixture but very weakly to the individual odorants (63.6 vs. 9.1 vs. 9.1%; AB vs. A or B: McNemar χ^2^ = 4.16, *p* < 0.05). Comparison of the responsiveness between the two groups clearly showed that AN-treated pups responded more to AB and less to A and to B than saline-treated neonates (χ^2^ > 5.49, *p* < 0.05).

Thus, when rabbit pups forgot the odors of the individual elements initially acquired during the conditioning to the AB mixture, their responsiveness to the mixture was extended over time. This result strongly supports our hypothesis that the learning of the mixture induced a competition between the memory of the AB configuration and the memory of the elements A and B.

## Discussion

The present results, obtained in rabbit pups using the AB weak configural mixture, demonstrate that the memory of odor elements is more robust and lasts longer than the configural memory of the mixture, and in fact can interfere with the maintenance of the configural memory. In the absence of elemental odor memory, the duration of the configural memory of the AB mixture is significantly enhanced. Although further work is required to determine whether these results generalize to other mixtures (weak configurally, configurally or, in contrast, elementally perceived mixtures), the present findings have important implications for understanding the mechanisms of odor mixture (and odor object) perception and the organization of odor mixture memory.

Advantage of elemental over configural memory of the AB mixture was first observed in the duration of memory following conditioning to the elements alone or to the mixture (Experiment 1). For example, conditioning to either element (A or B) alone induced a memory of that element that extended for at least 96 h. In contrast, conditioning to the binary mixture induced memory that extended no more than 72 h. Interestingly, the duration of memory for the mixture was age-dependent, while the duration of elemental memory was not. Animals conditioned to the mixture on day 1 reactivated memory for the mixture for 72 h, while animals conditioned on day 3 displayed mixture memory for no more than 48 h. Additional work is required to determine the mechanisms of this age-dependent variation in the duration of configural memory. Regarding general memory mechanisms, one may hypothesize that the information is more easily processed when the animal is exposed to a single odorant compared to a mixture of odorants. To be encoded correctly, a weak configural mixture could require a higher arousal level than individual odorants perceived separately, since the animals might have to share attentional level between the different stimuli of the mixture to engage associative learning for all stimuli (Sharot and Phelps, 2004). As a result, after only one conditioning session, the memory trace of a complex odor stimulus might be rather fragile and its decay might be faster over time in comparison with more simple stimuli. Here, the present conditioning was appetitive. One may therefore wonder whether similar results would be obtained with aversive conditioning, using odor-malaise association for instance (see Gruest et al., 2004), since a stronger arousal is then supposed to occur during acquisition. It would be of interest to evaluate in future experiments whether this other kind of conditioning induces longer lasting configural memory of the AB mixture.

Advantage of elemental over configural memory of odor mixture was also found after associative conditioning with the AB mixture, and within-subject testing to both the mixture and its elements (Experiments 2 and 3). After conditioning to this mixture known to be perceived configurally in humans (Le Berre et al., 2008; Barkat et al., 2012), rabbit pups expressed a weak configural memory of the mixture, meaning that they expressed both a memory for the mixture’s configuration and for the individual elements. In particular, they did not respond to AB at 96 h or 72 h (after conditioning on day 1 or 3, respectively) but still responded to A and to B at these retention times (Experiment 2). In terms of odor mixture processing, these results may seem surprising. Indeed, if the mixture is perceived in a weak configural way during the retention test, rabbit pups should perceive in the mixture both the elements they have previously learned and the AB configuration. Moreover, when pups have learned independently enough elements forming a mixture, they can respond (generalize) to the mixture even if the mixture is perceived in part configurally (Experiment 3a showing responsiveness to AB after successive learning of A and B; see also Coureaud et al., 2008). Therefore, after conditioning to AB on day 1 and because the pups responded to A and to B at 96 h, a response to the mixture could be expected. To explain the lack of response to AB at 96 h, we hypothesize that the AB mixture is, initially, weakly configurally perceived during the conditioning with the MP, but that a particular associative strength is then given to the configuration compared to the elements. As a consequence, the pups would perceive the mixture more as the configuration when they are exposed again to the mixture several days after conditioning. This hypothesis is supported by theoretical considerations suggesting that during conditioning to complex stimulus, a specific value is assigned to the configural representation (“unique cue”) independently of that given to each element (Rescorla, 1973; Rescorla et al., 1985). Alternatively, one may assume that during the conditioning, the presence of the MP leads rabbit neonates to perceive the AB mixture less configurally (i.e., weak configurally) compared to its processing in the absence of unconditioned stimulus (robust configural perception).

Importantly, the present results suggest that the long-term memory of the elemental and configural representations of the AB mixture compete. That is, with intact elemental memory, configural memory of the mixture degrades significantly faster than memory of the elements (Experiment 2). In contrast, if elemental memory is disrupted by selective reconsolidation blockade, configural memory of the mixture is maintained significantly longer (Experiment 3b). This indicates that removing a potential source of interference facilitates memory performance for the AB configural information. The retrieved memory of the AB mixture can thus be regarded as the sum of conflicting processes involving, in our case, the elemental memory and the configural memory. The outcome seems dependent on the dominance of one of the memories at the time of retrieval, here the elemental memory, a result that contributes to unravel aspects of memory organization (e.g., Eisenberg et al., 2003; Suzuki et al., 2004; Bradfield and Balleine, 2013). The competition would rely on the retention time (Experiments 1 and 2) and the simultaneous (but not successive; Experiment 3a) exposure to odorants forming a weak configurally perceived mixture. This interference/competition may happen theoretically and first during consolidation of configural and elemental memories of the mixture. However, this assumption has certainly to be ruled out, since we have previously shown in newborn rabbits that consolidation of odor stimuli learned by association with the MP happened in the first 4 h after conditioning (Coureaud et al., 2009b); the consolidation phase is thus terminated well before the erasure of A and B memories performed here (i.e., 24 h after conditioning; Experiment 3b). Therefore, in the present paradigm, interference/competition between configural and elemental memories of the mixture certainly occurs during maintenance and/or recall of the memories rather than during consolidation phase, with elemental memory the stronger of the two. Even if the most probable explanation for the absence of response to AB at 96 h is the absence of recognition of the AB configuration, one can not exclude the possibility that rabbit pups still find familiarity in AB but without any motivational significance.

The present results do not allow direct determination of the mechanisms of elemental and configural memory interaction, although hypotheses based on the known neurobiology of odor mixture processing can be developed. In rodents, odor mixtures are processed differently by the olfactory bulb and primary olfactory (piriform) cortex (Wilson and Sullivan, 2011). For example, the activity of olfactory bulb mitral/tufted cell neural ensembles is strongly consistent with pattern separation processing–responding uniquely to even minor changes in mixture elements (Barnes et al., 2008; Chapuis and Wilson, 2011; Sahay et al., 2011). Thus, it has been hypothesized that olfactory bulb neural ensembles respond to odor mixture features or components, rather than to the mixture configuration. In strong contrast, piriform cortical neural ensembles respond in a manner consistent with configural processing. In fact, even at the single-unit level of the piriform cortex, individual cells can distinguish between mixtures and their components in cross-adaptation procedures (Wilson, 2003). Mitral/tufted cells, on the other hand, show strong cross-adaptation between mixtures and their components, again consistent with an elemental or feature-dependent process within the olfactory bulb (Wilson, 2003). After having reported in newborn rabbits mapping of activations induced by the MP or by a MP-learned odorant in the olfactory bulb and central regions, including the piriform cortex (Charra et al., 2012, 2013) we are currently assessing the brain activity mapping of the AB mixture (Schneider et al., in preparation).

Odor memory is dependent on plasticity within both the olfactory bulb and piriform cortex (Wilson et al., 2004), even in neonates (Moriceau and Sullivan, 2005; Charra et al., 2013; Fontaine et al., 2013). In the context of the present results, and waiting for further results obtained with the AB mixture as with other odor mixtures, we hypothesize that the more stable elemental memory of an odor mixture may mainly depend on olfactory bulb plasticity, while configural memory of the same mixture may mainly depend on piriform cortical processes (in accordance with the presumed role of piriform cortex as an associative cortex; Johnson et al., 2000). These cortical processes may be less robust during early development than memory-dependent events in the olfactory bulb, resulting in the distinction between these two memory forms in the present study. The less robust configural memory might depend on differences in the developmental program of both the olfactory bulb and piriform cortex: functional odor maps in the glomerular layer seems rather well defined after birth (Guthrie and Gall, 1995) whereas the maturation of inhibitory processes and the intracortical associational fibers throughout the 3-layered piriform cortex (Schwob and Price, 1984) might follow a slightly different time curve (Garske et al., 2013). One may also suggest that this distinction does not rely on robustness of cortical processing only, but on changes during animal development in the dialogue between sensory/mnesic regions of the brain (olfactory bulb/piriform cortex/amygdala/hippocampus), in accordance with the behavioral and adaptive needs of the animal (Schacher and Hu, 2014). Future work could combine the powerful reconsolidation-mediated dissection of memory utilized here, with neurophysiological techniques to explore neurobiological underpinnings of these basic memory and perceptual processes.

Finally, the present findings are further evidence that while odor mixtures are perceived sometimes configurally, and conscious analysis of odor mixtures is notoriously difficult (e.g., Jinks and Laing, 1999), information about the underlying individual components can remain intact in both neonatal rabbits (shown here) and adult humans (Grabenhorst et al., 2007). In the case of newborn rabbits, this may be especially important given the life and death importance of odor recognition to interact with the mother, attach to the nipples, survive and grow up. Having access to both the elements and the configuration may help ensure successful recognition, improved discrimination between odorous substrates or conspecifics in the surroundings, adaptation to the actual environment or anticipation of social and feeding changes that will come later in development.

## Conflict of interest statement

The authors declare that the research was conducted in the absence of any commercial or financial relationships that could be construed as a potential conflict of interest.
